# Supplementary material for: Mapping of schistosomiasis and soil-transmitted helminthiases across 15 provinces of Angola
Source: PLoS Negl Trop Dis. 2022 Jun 30;16(6):e0010458. doi: 10.1371/journal.pntd.0010458 (PMC9278740; doi:10.1371/journal.pntd.0010458)
Supplement: Attachment — Submitted filename: Response letter.docx [file pntd.0010458.s005.docx]

**Response Letter**

Luanda, 18^th^ of October 2021

**To Plos NTD Deputy Editor**

Christine M. Budke

**Subject:** Review of the Submitted Article “Mapping of Schistosomiasis and Soil-Transmitted Helminths across 15 provinces of Angola”

Dear Christine,

In response to your communication from September 9^th^ 2021, on behalf of the co-authors I am uploading a revised version of the referred manuscript with the following key changes:

1. Inclusion of author summary as suggested
2. Inclusion of Introduction section
3. Inclusion of a list of legends for additional files
4. Inclusion of tables in the body of the manuscript
5. Inclusion of the email contact of the National Ethics Committee of Angola

Please note the following answer for one of the points raised:

- We note that the following authors have not yet confirmed their authorship on this manuscript: Ricardo Thomson. **Answer:** Please be aware that Dr. Thomson died during the manuscript writing and for obvious reasons he cannot confirm authorship. Please let us know how to proceed about this author. We have reached out the other authors who should, by now, have confirmed authorship

Thank you for your revision and suggestions.

On behalf of the authors,

Sérgio Lopes, RN, MSc
