# Supplementary material for: Mapping of schistosomiasis and soil-transmitted helminthiases across 15 provinces of Angola
Source: PLoS Negl Trop Dis. 2022 Jun 30;16(6):e0010458. doi: 10.1371/journal.pntd.0010458 (PMC9278740; doi:10.1371/journal.pntd.0010458)
Supplement: Attachment — Submitted filename: pntd-d-21-01157-r1-reviewer.docx [file pntd.0010458.s006.docx]

**Title and Author section**

**L2:** in the title**;** Mapping of Schistosomiasis and Soil-Transmitted Helminths across 15 provinces of Angola, the terms **s**chistosomiasis and **s**oil-**t**ransmitted helminths/**iases** in the main tile should be written without capitalization as indicated.

The same format should be followed for the rest of the article except where it begins a sentence. Also consider the difference between helminths and helminthiases in the context of the survey objective. The two are quite different as *helminths refer to the parasites themselves but helminthiases refer to the infections caused by helminths.*

***L15:*** *E****x****panded, check spelling and correct.*

**Article Main Body**

**L1: Abstract**

**L3:** write **s**chistosomiasis and **s**oil-**t**ransmitted helminths/**iases** in the main tile without capitalization. The same should be done in the rest of the article except where the terms begin a sentence.

**Key Words:** Authors should not the difference between words and phrase in this section, use appropriate punctuation separating words.

**Full and Short title:** The short title especially should be further abridged to fit the definition of a short title eg, *Mapping of* ***s****chistosomiasis and* ***s****oil-****t****ransmitted* ***h****elminths/* ***helminthiases*** *in Angola.*

**L8: Methods**

**L12:** Replace **Faecal** with more conventional term **stool,** better begin a sentence with a **word** instead of number of %, rewrite the sentence.

L15: is this word **Norte** a noun of correct English **work**

**L17: Results**

L19: be more uniform in expressions related to prevalence, compared to risk; high, moderate, low…, secondly does the level of prevalence as high, medium, and low equate the same as risk?

**L20-L22.** Correct the spelling of the word prev**e**l**a**nce ***to prevalence.***

*What is the mean national prevalence of schistosomiasis and soil-transmitted helminthiases for boys and girls?*

**L29: Conclusion**

**L30:** Provides/provided/generated **NB. The exercise is concluded and is in the past tense.**

***L1:* Author Summary**

**L4:** be consistent throughout the document in the way you inscribe **s**oil**-t**ransmitted **h**elminthias**e**s compared to Soil Transmitted Helminthiasis

***L5/L6:*** *remove capitalization mass drug administration and campaign(s) as written, the first mention of the term can be followed by (MDA) as shown in the bracket. Subsequently, the abbreviation may be widely used than the full phrase.*

***L6:*** *campaign****s*** ***is/are***

***L7:*** *watch out for capitalizations and consider alternative use of shorter abbreviations like SCH/STH.*

***L10:*** *Be specific about the number of parasitological analyses, and also watch this (.)carefully nearly 13****.****000*

***L12&L13:*** *be consistent in the choice in the use of the terms* ***mapped*** *and* ***surveyed*** *when referring to the same activity, and their meaning should be clarified if any difference exists.*

***L1: Introduction***

***L20:*** *What is Bie; district, or town or province etc?*

***L20:*** *is* Benguela and Luanda provinces? Be consisted as indicated in Malange and Huila provinces…L22-23.

World Health Organization (WHO) road map for neglected tropical diseases 2021-2030^1^

***L1: Methods***

***L2: Study Design***

***L9:*** indicate the total number of participants studied nationally/interviewed, and their proportion by sex.

**L11: School Selection Procedure**

***L12:*** *what category of schools were the study participants selected from, primary, secondary, or both.*

**L26: Study Participants**

**L29:** how exactly was the systematic random selection done, which skip intervals were used, how was biased controlled during the process?

**L33:** check spelling of **children** (‘s).

**Table 1:** It would be informative to include indicate at province level, the distribution of males and females and mean age by province

**Table 2 and 3:** The titles should be reviewed to befit the information displayed in the table;

**General Comment:** Line numbers have been set to restart each page and needs to be **continuous**. This makes the review process quite complicated at this point.
