# Supplementary material for: Mapping of schistosomiasis and soil-transmitted helminthiases across 15 provinces of Angola
Source: PLoS Negl Trop Dis. 2022 Jun 30;16(6):e0010458. doi: 10.1371/journal.pntd.0010458 (PMC9278740; doi:10.1371/journal.pntd.0010458)
Supplement: Attachment — Submitted filename: 20220214_Response to reviewers.docx [file pntd.0010458.s007.docx]

Luanda, February 14^th^ 2022

Dear Editor of PLoS NTD,

Thank you for sending the reviewers’ comments on our paper “Mapping of schistosomiasis and soil-transmitted helminthiases across 15 provinces of Angola”, PNTD-D-21-01157R1.

Please find below a response to the suggested revisions, indicating changes made in the manuscript.

**Methods**
Reviewer #2:
The data availability statement is incorrect because when it says where can the data be found, the authors have put these data cannot be shared.

We have amended that statement to make data available upon request. As per journal request, data is made available by a third independent party, in this case National Institute of for Health Research (INIS in its Portuguese acronym).

Line 11 of the abstract says 31,938 children in the study but in line 10 of the author summary says 13.000 which I presume is supposed to be 13,000 but is also inconsistent with the abstract or unclear if the correct number – what is the almost 32,000 if not the number of children included? In future do not restart page numbers for each section of the document. It makes referencing the correct part difficult, especially seeing as you have no page numbers. We apologize for this major inconvenience for your review. We have kept it for reference and to track changes made but we will certainly take that on board in the future. We have corrected the number in the summary that was mistakenly placed. Thank you for pointing that out.

Introduction Line 7 – it is also to do with water contact patterns, sociodemographic factors etc, these all contribute to the force of infection.

Thank you for raising this important point. We have readjusted the sentence to include the factors you point out and others that are known to be of relevance for the force of infection

Line 13 – you have referred to these as abbreviations above so SCH and STH should be abbreviations consistently. Also, schistosomiasis is the disease so does not need to be capitalised, but Schistosoma haematobium on line 19 has not been taxonomically declared before and should in this instance be spelt out with a capitalised S.

We have changed accordingly across the whole paper. Thanks for noting this.

Lines 24-25 – What do you mean by this sentence? All prevalence/ burden measures in any location are estimates as nowhere is the whole community sampled.

We have adjusted the language and hope it is clear now that what we meant to say was that a survey confirmed the high prevalence previously reported.

Line 7 page 2 of your introduction – is MENTOR an acronym?

Yes. This has been added to the acronym list

The introduction would benefit from reference to the maps. It is quite listy in terms of locations and previous mapping efforts. Perhaps in the introduction, some guidance from the authors for the readers as to whether the overall prevalence and intensities of infections for schistosomiasis or STH are going up or down? Why is this mapping effort so important? As it stands, it seems like there have been regular and ongoing efforts to map these diseases.

We have made a reference in the introduction to the type of mapping and its importance and the previous studies (not wide mapping exercises conducted). Since the previous studies only refer to small scale scale studies, we have clarified that in the intro as well. For that reason, it is not possible to compare prevalence and make the required statements about prevalence increase/decrease

Line 20 School selection criteria – close not closed?

Amended. Thanks for the suggestion

Line 24 School selection criteria – some clarification for why you wanted to oversample in these locations would be beneficial.

This is based on WHO protocol and the existing understanding that Schistosomiasis is highly associated with proximity to freshwater bodies. We added a sentence and a reference to help clarify

Line 10 Parasitological diagnosis – mansoni should not be capitalised. It has also not been taxonomically described yet in the text and should be fully spelt out in this instance.

Changed. Thanks for the correction

Line 15 Parasitological diagnosis – this is not true. The Kato-Katz method lacks sensitivity, egg excretion from the human host is highly over dispersed and does not necessarily reflect the density of female worms – there are a few hypotheses for this including density dependent reproduction, host acquired immunity and worm senescence. As it is essentially incorrect, please remove this sentence. Further to this, you do not explain at all how many stool samples you use, or over how many days (Lamberton, 2014). The methods generally lack any useful detail and give too much opinion.

We appreciate your comments. We have removed the mentioned sentence. While we wanted to make reference to the intensity of infection, we realize the sentence does not accurately reflect the initial idea. We also clarified the number of samples used. Please note this is an operational mapping exercise aiming to map an entire country, with obvious limited resources. The principle objective is – as stated in the draft paper - to quantify the burden and distribution of these diseases across fifteen provinces in Angola in order to be able to adequately plan preventive chemotherapy interventions. The objective is quite different from the one you have suggested which, from our reading aims to a) compare the accuracy of the currently available POC-CCA and one to six Kato-Katzs; b) evaluate the epidemiological implications of diagnostic methods for control program M&E and praziquantel and efficacy studies and c) Inform on the number of Kato-Katzs required to accurately detect S. mansoni infections pre- and post-praziquantel treatment and whether POC-CCAs are suitable alternatives. While we recognize the importance and thye accurace of longitudinal methods to improve diagnosis accuracy, it should be recognized that a country wide mapping that included nearly 32.000 children would be operational unfeasible and financially impossible to implement. For this reason, while recognizing that methods used may not be fully accurate (we added a sentence in the discussion recognizing that), the use of standard protocols recommended by WHO that have been used across SCH and STH endemic settings for the same objective provide an accurate estimate of the prevalence of these diseases to inform subsequent MDA campaign allocation.

Lines 24-33 are superfluous. If you really want to include them put them in an appendix but for the purpose of the main text I would remove them.
We appreciate your suggestion. We have removed those as we agree they provide mainly operational information of how the mapping was implemented.

Lines 1-9 ESPEN data collection – Rather than describing what the ESPEN tool is, I would like to understand clearly and concisely what it was used to collect. This is not clear from the text. This can be significantly streamlined.

Thanks for this very important comment. We recognise the text was too much focused on the app itself and not on the variables that were collected. We significantly changed this section to integrate the details collected by ESPEN Collect.

Line 21 Statistical Analysis – you have a (ref) that I presume should be a reference.

Thank you for picking up on this, this has now been corrected in the manuscript.

Line 30 Statistical Analysis – when you say clustering here, do you mean that the school was considered as a random effect in the model? Does this mean you did no analysis on infection intensity despite having all of these egg counts? I think this is a really important point. You could have high prevalence of low intensity infections. Furthermore, we know that the KK method performs poorly in low prevalence and low infection intensity settings – without an analysis on the intensities how can we really tell if this mapping is reflective of true infection distribution? I’d be likely to think there are more infections than this has detected if the intensities are low and maybe more likely to think it has done a good job at characterising infections if the counts are very high. With such huge sample sizes I am also unsure why you needed to do a univariate analysis first. This method notoriously produces spurious results. Did you not include any interactions or random effects
either? I feel the statistical analysis here is either lacking effort and rigour or maybe lacking a more substantial technical description? I think I will not understand which it is until at least a more detailed technical description of the analysis is provided. Indeed, if this is such an important mapping effort, then peoples quality of life can depend on these results meaning statistical rigour is essential. Also, I wonder what the malaria treatment situation is given the prevalence of malaria in Angola and that malaria treatment has anti-schistosomal effects. “Data” is a plural word so reporting should be “no data were” or “not data are” rather than “no data was” and “no data is”.

Thank you for your comments. The primary aim of this study was to map the prevalence of SCH and STH infection at the municipality level across the 15 provinces in Angola. In a secondary objective we explored whether the presence of a latrine or water source in the school reduced the likelihood of a SCH/STH infection, respectively. We have now clarified that this was a secondary aim, and therefore adapted the tables to reflect this.

Due to the sampling methodology, we believed it was important to account for the clustering at the school level by using robust standard errors. We believe this was sufficient as we would be adding province as a fixed effect in the multivariable model. Given the exploratory nature of this second objective, we believe this approach to be appropriate.

We have now adapted to the manuscript to ensure the word data has been used appropriately.

Discussion line 21 – do you think communication is needed for impoverished children to wear shoes or do you think this is a funding issue where funds should be diverted to enable shoe wearing? I think you really need to discuss the fact that egg-based diagnostics, particularly for SCH, is simply not an adequate tool for capturing true prevalence and intensity measures. This is not mentioned at all in your discussion and is a major problem. Given that there is no description for how many counts per stool over how many days, I am presuming also that just one count was done. This is also inadequate and grossly underestimates prevalence and infection intensity. These are major limiting factors to the credibility of the estimates provided. Also, in the context of the new WHO roadmap, what is the part played by animals in the maintenance of S. haem? Given recent work that has been published (Borlase, 2021 Leger, 2020) this deserves a comment in the discussion. You also have no comment of the fact that you only sampled children. Adults can harbour incredible infections and manifest chronic morbidity more often. In such instances then, who is really suffering the most from infection, children with high egg shedding rates, or adults with low egg shedding rates but high levels of morbidity (caused by the eggs not shed). you say that the WHO give guidance for taking district level estimates and turning them into subdistrict estimates. What is this method?

We point out for the main objective of the study. While the methods may have several limitations – which we now addressed in the discussion section – the methods used are not new, nor inadequate for the aim it is purposed. Otherwise, MDA campaigns supported by WHO across several countries would be grossly missing its target. A mapping of this kind should also be adjusted to the country reality. This is the first ever country mapping done at this scale in Angola and while it would have been fantastic to survey animals, adults, conduct estimates over time with several stool samples, the cost of doing it would give us a super accurate estimates but probably would be possible to do it in a single province, leaving all the others without any information to plan MDA and start implementing chemoprophylaxis interventions. Angola has been completely neglected in terms of NTD control interventions. The mapping exercise is the first step towards some specific work and as much as we would love to implement a very solid mapping exercise, the resources and capacity do not exist in country. We had to adjust the protocol and procedures to existing field reality to have – as accurate as they can be – prevalence estimates.

We have addressed the limitations you have shared in the limitations. The WHO guidance can be found here (<https://espen.afro.who.int/system/files/content/resources/Report%20of%20the%20Workshop%20on%20SCH%20sub-district%20level%20data%20review%20for%20shrinking%20the%20map.pdf> ) which is the same reference listed in the reference list.

Reviewer #3: Authors made a good attempt in investigating a very important issue on evidence guided decision making. however, further clarity on the how the results reflect on the objective is required. In terms of the results, authors needs to bring out variate analysis output gender and age variations especially at province levels.
The sample size is sufficient enough for this types of surveys an perceived adequate to prove useful information.
No ethical issues of significance were noted, the authors however needs to demonstrate coherently how consent have been given, written, or signed, could the parents read, or who was available to explain to parents to seek consent, does consent seeking involve translations. was there any participants excluded for reasons of non consent.
The objective of this survey, which is stated as "mapping the epidemiology of SCH and STH" should be defined, to give give a grasp of what the authors meant by mapping the epidemiology of SCH and STH.

Thank you for your comments. We have clarified the objective to align the description in the abstract with the description in the main body of the text. The analysis was also adjusted for gender and province and we added further details on how consent forms were handled.

**Results**
Reviewer #3: Table 1, 2 and 3, need improvement on the appropriate titles, and content. Table 1 could incorporate more information on demographics of the study participants at province or municipal levels.

Thank you for your suggestion. Due to the sampling method, the median/IQR of children included was the same across all provinces. However, we have now supplemented Table 1 to show the sex distribution of children recruited at the province level.

The presentation of the results and the discussion should relate to the objective including how it will guide treatment arrangements.
How does the integrated treatment relate to or affect the wider community based MDA is not very clear.
Thank you for this comment. We recognize the relation between the mapping and the treatment plans is not addressed in the results because we wanted to expand broader to the overall NTD Master Plan for Angola. For that reason, we have expanded on that in the Discussion Section, on paragraph 6, where we explain the treatment needs that arise from this mapping exercise. We added a sentence about the need to integrate efforts with community MDA. However, LF and Onco prevalence estimates are outdated and treatment at community level is not currently being implemented.

**Conclusions**
Reviewer #3: Authors should be concise but conclusive on the final take on the extent in which the objectives of the study had been achieved and how it will be used to guide strategic prevention and control strategies.
The public health relevance of the study had been achieved in practice. Although the information presented should be more coherent in both writeup style, arrangement and flow of the information being presented and expressions.

We have made some changes to adjust writeup style throughout the paper and expect this make it more readable. We have also improved the final conclusion statement to clearly address how objectives of the study have been achieved.

**Editorial and Data Presentation Modifications**
Use this section for editorial suggestions as well as relatively minor modifications of existing data that would enhance clarity. If the only modifications needed are minor and/or editorial, you may wish to recommend “Minor Revision” or “Accept”.

Reviewer #1:

Page 9, line 3 – “individuals’” should be “individual’s”. Changed
Throughout: “S. Haematobium” should be “S. haematobium”. Same with S. Mansoni. Changed
Throughout: “Schistosomiasis” should be “schistosomiasis” unless at the start of a sentence. Changed
Page 9, line 35, recommend changing “global” to “nationwide”. Changed
Page 10, line 4: “remainder” should probably be “remaining” Changed
Page 10, line 7: is “municipalities” here the same as “districts”? We have harmonized the language to have only district as denomination
Page 14, lines 5-6: “Informed Consent forms were sought near participants parents” should probably be “Informed Consent forms were sought from participants’ parents”. Changed
Page 14, line 6: “Team elements” might be better as “Team members” or “Team leaders”. Changed
Page 16, lines 21 and 22: “pavement” might be better as “paved”. Changed
Page 20, line 34: “age’ could be removed. Changed
Page 21, line 3: “prevalence sub-district level” should probably be “prevalence at the sub-district level” Changed

Reviewer #3: Line numbering was set by the authors to be repetitive per page and made review especially making reference to specific lines very cumbersome. Authors should set line numbers to be continuous instead.

As stated for other reviewer, we really apologize for the major inconvenience. We recognize that it may have been quite hard for revising and we acknowledge it will be also very hard to track the changes made in this review. Please accept our sincerest apologies for this.

**Summary and General Comments**

Reviewer #1: This is a well written and very interesting paper looking at the distribution of SCH and STH in Angola. The sample size is extensive – stretching to 640 schools, 32,000 children across 131 districts. These data will clearly be of use in planning SCH and STH interventions in the country and as a staging post to 2030 targets.
A few questions outlined below, most are minor.

Comments
Abstract – results. When saying infection was related to age and sex, I’d recommend making clear in what direction. E.g. older people more likely to be infected. Males more likely to be infected. This is included in the results section, add to abstract.

In light of other reviewers comments, we have revised the analysis and removed this reference from the abstract as it would require several adjustment explanation to accurately reflect the results.
Abstract – mention whether you are referring to S.mansoni or S.haematobium.

Note that in the abstract we refer to any SCH infection. This has been clarified.
Author summary – this mentioned a sample size of 13,000 children, but 32,000 are referenced in the abstract. Which is accurate?

We have now corrected the figures that should reflect the total of 32.000 sampled.
Methods: For children indicating that they had taken parasitic drugs in the last six months, how many were there?

That information was not recorded because no MDA were being conducted in any of the provinces. So, while that was set as an exclusion factor that was asked in every school, it turned, we rapidly realized that this criterion did not apply.

Methods: “Schools selected that were closed to each other were purposively replaced by schools in locations known to be in areas of increased risk for Schistosomiasis transmission.” I think that’s a fine way to proceed, but we should be aware that it doesn’t give rise to unbiased estimates of prevalence.

Correct and agreed. Please note that this is a recognized standard for this type of mapping as per WHO procedures. This was done mostly when in urban settings where concentration of schools is higher. As stated in WHO guidelines for mapping, “simple” PPS strategies may fail to identify hotspots of transmission of SCH as they happen in very focal areas close to recognized ecological settings (freshwater habitats where snails live).

Methods: Student behavior questionnaire: agree that if data are not robust enough, they should not be used for analysis. What is the reason for that – due to students not understanding the questions? Methodological flaws?

That’s a key limitation we cannot fully justify and is addressed as such in the limitations sections. We believe it is a mix of both. On one side, students may have missed the point of the questions and provided biased/socially acceptable answers (about sanitation behaviours for example) but we also found many missing entries point out for flaws in data collection. As a team, we assessed that to collect these specific elements, a dedicated team member should be nominated so that questionnaire could be adequately implemented.

Methods: For age, how is the odds ratio calculated. For each additional year of age? Or comparing age categories?

Odds ratios were calculated for each year increase in age. However, due to streamlining the objectives of the analysis, these tables have now been adapted.

Page 16, lines 29-31. Interesting that children were less likely to have SCH infection if they had STH infection (but not in the multivariate analysis).  Why do the authors think that is? Upon clarification of our secondary objective, these analyses have now been removed to focus on our variables of interest: presence of latrine/water source in the school.

Page 20, lines 17-19: “Overall, data indicate that WASH in school investments are essential to provide essential infrastructure and to foster its use and the adoption of healthy sanitation behaviours”. I agree that there are so many reasons why school WASH infrastructure should be improved. But this paper does not show SCH / STH infection to be one of them.
That’s a very valid point. Thank you for raising this. In fact, the data did not show objectively that need and that was an overstatement from our side. We have adjusted that sentence to accurately reflect what is shown across the paper.

Could consider bringing Additional File 5 (number of districts in each province to be targeted for MDA) into main body. But not compulsory.

We have considered that option as well, but there are editing limitations (number of tables) to do that.

Reviewer #3: This survey is an important activity as part of the NTD elimination roadmap, and information generated will be very useful in guiding efforts in intervention. What the authors need is review the writing style, grammar, expression, flow and other typographical errors. Analysis and results should be more extensive in portraying province and municipality variance for ages and sexes and the strategy for treatment and other intervention like WASH, on the basis of evidence of level of prevalence high, medium and low.

We have revised the paper and hopefully have improved writing style, grammar, expression, flow and other typographical errors. Please refer to all changes done in results and discussion section as well as we hope they provide the needed expansion to some of the gaps existing in the original paper.

Yours sincerely,

Sergio Lopes

On behalf of all authors
