# Supplementary material for: Mapping of schistosomiasis and soil-transmitted helminthiases across 15 provinces of Angola
Source: PLoS Negl Trop Dis. 2022 Jun 30;16(6):e0010458. doi: 10.1371/journal.pntd.0010458 (PMC9278740; doi:10.1371/journal.pntd.0010458)
Supplement: Attachment — Submitted filename: ReviewerComments-PNTD-D-21-01157-R2.docx [file pntd.0010458.s008.docx]

**QUESTIONS AND COMMENTS FOR THE AUTHORS**

1. If a nationwide survey was carried out in 2005, how did the same or a similar one in 2014, 9 years later became a baseline survey. Please clarify.
2. Countrywide **mapping exercise** in 2018 and 2019, to quantify the burden and distribution. In another statement it was from 2018 to 2019. This portrays different meaning and is understood differently. Secondly there should be consistency in the use of the term (mapping, survey, exercise etc) meant to describe the activity in question.
3. The authors stated that from July to December 2019, a cross sectional survey was implemented in 14 remaining provinces- this however contradicts the earlier statement that a nationwide survey carried out in 2005.
4. Some of the additional files especially 1 and 2 which contain data on the distribution and prevalence of SCH and STH should be incorporated in the article as tables. Also authors may equally consider the same for the table on MDA strategy.
