# Supplementary material for: Mapping of schistosomiasis and soil-transmitted helminthiases across 15 provinces of Angola
Source: PLoS Negl Trop Dis. 2022 Jun 30;16(6):e0010458. doi: 10.1371/journal.pntd.0010458 (PMC9278740; doi:10.1371/journal.pntd.0010458)
Supplement: Attachment — Submitted filename: 20220424_Response to reviewers.docx [file pntd.0010458.s009.docx]

Luanda, April 24^th^ 2022

Dear Editor of PLoS NTD,

Thank you for sending the reviewers’ comments on our paper “Mapping of schistosomiasis and soil-transmitted helminthiases across 15 provinces of Angola”, PNTD-D-21-01157R1.

Please find below a response to the suggested revisions, indicating changes made in the manuscript.

Comments From R2:

1.	If a nationwide survey was carried out in 2005, how did the same or a similar one in 2014, 9 years later became a baseline survey. Please clarify.

Thank you for noting this very important point that for the authors may sound minor but may generate some confusion for the reader who may not be aware of NTD background and work in Angola. To clarify, the 2005 survey was based on ecological regions with limited number of schools sampled by region and with results from few schools mapped in a region extrapolated to wide geographic areas. Methods were not in line with current mapping protocols defined by WHO which limited the targeting of interventions at district and sub-district level. MDA allocation was done to wide geographical areas. The 2014 baseline mapping in 3 provinces, followed by the mapping reported in this paper further confirmed differences in prevalence of these diseases at district and sub-district level that were not previously captured by 2005 data. We have tried to clarify it in the manuscript so that differences in methods are clear.

2.	Countrywide mapping exercise in 2018 and 2019, to quantify the burden and distribution. In another statement it was from 2018 to 2019. This portrays different meaning and is understood differently. Secondly there should be consistency in the use of the term (mapping, survey, exercise etc) meant to describe the activity in question.

Thank you for this comment. We have tried to clarify the dates and the geographical implementation in each of the periods used for this mapping. We hope this is clearer now. As for the use of different terms, we have tried to make it consistent were mapping refers to the overall study design and implementation whereas the use of the term survey is used more to define the methodological details. It should be noted that in some cases, it is hard to use the same term repeatedly for reading purposes as it can become fastidious for the reader. We hope changes made help improving the paper in regards to the consistency in use of terms.

3.	The authors stated that from July to December 2019, a cross sectional survey was implemented in 14 remaining provinces- this however contradicts the earlier statement that a nationwide survey carried out in 2005.

Thanks for noting this. We have now clarified the periods of implementation and the geographical scope of implementation.

4.	Some of the additional files especially 1 and 2 which contain data on the distribution and prevalence of SCH and STH should be incorporated in the article as tables. Also authors may equally consider the same for the table on MDA strategy.

The authors fully subscribe your comment. The tables mentioned are of extreme importance to understand the distribution of diseases mapped at district level. However, the editorial manager recommended that Additional Files 1 and 2 should not be included as they are tables with 150 rows each, which would not display well in the body of the article. Besides, the maps provided somehow display the same information (even though based on intervals and not on the point estimated as the tables). For these reasons, we opted to keep those two tables out of the main body of the text. We have included the MDA table strategy as we agree it is quite important to showcase the practical implications of this mapping. The additional file 5 is now in the main body of the text as Table 3.

**Methods**
Reviewer #3: I am of the opinion the authors should limit the objective to the distribution of the said diseases, something like the prevalence and distribution. The term burden seems more complex to be established and seems inadequately addressed by the data presented.

Thank you for this comment. We have amended the objective scope accordingly. We agree that the scope of this study and data presented cannot be used to extrapolate to the burden of the diseases. As you correctly mention, the use of the term burden is complex as it entails the impacts of the disease beyond prevalence which is what this study is measuring.

**Summary and General Comments**

Reviewer #2: This is the second time I have handled this manuscript. Given the satisfactory response to the original review I just have a few additional minor things:
First, I totally appreciate the amount of effort that has gone into this mapping. My commentary on the lack of sensitivity with a single Kato-Katz was not a slight on your effort but on the continued recommendation from large bodies such as WHO, for its use, and thus a continued lack of financial support provisioned for improved diagnostics and labour. The 2030 goal is ambitious and if we cannot truly understand the dynamics how will it be possible to reach. As such, I hoped to see in the original version, a more thorough discussion of this as this is where experts such as yourselves can have your say and highlight this dire need. Indeed you have responded to my request for more of this in the discussion with a small section but I stand by my original review comment - that there are additional complexities to control that the WHO mapping protocol just does not capture. I do not expect you to add more to the discussion at this point, the manuscript is fine as it is, but just
food for thought.

Thanks once again for your very thoughtful comments. We fully subscribe your point of view and tried to add more on the previous review into the diagnostics discussion as, like you said, better and more sensitive tools are needed to effectively meet the 2030 targets. We thank you for your initial comment as it really helped improving the current version and improve discussion of one of this paper limitations and – we risk saying – a general limitation of currently used tools for mapping exercises. Thank you for that.

Line 17 Parasitological diagnosis - remove the fullstop after Schistosoma

This has been corrected now. Thanks for spotting it.

Line 23 ESPEN collect section - Expanded not Espanded

We have checked this across the article and believe it is now corrected.

Yours sincerely,

Sergio Lopes

On behalf of all authors
